# Considerations on the Clinical Relevance of Climatic Differences in *Pseudomonas aeruginosa* Rates in Diabetic Foot Infection

**DOI:** 10.1093/ofid/ofae724

**Published:** 2024-12-11

**Authors:** Hana Niino, Shunkichi Ikegaki, Kentaro Iwata

**Affiliations:** Integrated Clinical Education Center, Kobe University Hospital, Kobe, Japan; Division of Infectious Disease Therapeutics, Department of Microbiology and Infectious Diseases, Kobe University Graduate School of Medicine, Kobe, Japan; Division of Infectious Disease Therapeutics, Department of Microbiology and Infectious Diseases, Kobe University Graduate School of Medicine, Kobe, Japan

To the  Editor—We read with great interest the article by Winski et al., which reported significant variations in the prevalence of *Pseudomonas aeruginosa* in diabetic foot infections across different US climates [1]. This information provided a better understanding of diabetic foot infections, but we would like to raise two concerns regarding their findings.

First, Winski et al. did not put race into the multivariate logistic regression analysis as one of the covariates. There are significant racial differences in patient demographics depending on the climate. The proportion of non-White patients was only 11.8% in the cool dry climate and but was 33.2% in the hot humid climate. Racial differences might affect the causative organisms of infections; a study found that Hispanic patients with cystic fibrosis have an increased risk of acquiring *P. aeruginosa* compared with non-Hispanic patients [2]. In addition, racial differences could affect financial background, basic hygienic environment, and control of diabetes. We wonder why this was not included in the multivariate analysis as a covariate.

Second, we would like to question the clinical significance of the difference in *P. aeruginosa* prevalence (6.2%–11.6%). While statistically significant, a difference of ∼5% may not be sufficient to change clinical decisions on antibiotic selection. Furthermore, the true rate of *P. aeruginosa* could be even lower because superficial swab cultures, which are less specific [3], were not explicitly excluded, allowing colonization cases to be included in the analysis. Even in hot humid climates, empirical treatment may not be necessary unless the conditions are severe, and the opposite is also true: Empirical coverage of *P. aeruginosa* will be necessary in cool climates if the patient’s condition is severe enough. We find that the results of Winski et al. suggest that climate conditions little serve in determining empirical antibiotic selection in diabetic foot infections.
